# Supplementary material for: Identification of Invariant Sensorimotor Structures as a Prerequisite for the Discovery of Objects
Source: Front Robot AI. 2018 Jun 25;5:70. doi: 10.3389/frobt.2018.00070 (PMC7806078; doi:10.3389/frobt.2018.00070)
Supplement: Supplementary file 1 [file Presentation_1.PDF]

Supplementary Material: Identification of Stable Sensorimotor Structures as a Prerequisite for the Discovery of Objects

1 SUPPLEMENTARY DATA

In this supplementary information section we present the results of the simulation in alternative configurations, motivated by questions of by the reviewers.

1.1 Influence of the hyperparameters of the simulation

We first assess the robustness of our model to changes in the simulation hyperparameters. We briefly analyze 4 hyperparameters: the number of exploration steps performed by the agent, the size of the proto-objects, the size of the environment, and the number of centroids in the $k-means$ clustering step. The results globally show that our model is robust to reasonably small changes in these parameters.

1.1.1 Influence of number of time steps

In the experiment presented in the paper, $3e7$ time steps were performed by the agent. Here, we investigate the results of the simulation when the number of time steps is reduced to $5e6$ and $1e6$ steps. The results are shown in Figure S1. When the agent performs $5e6$ exploration steps, the proto-objects are well extracted. However, with $1e6$ time steps, the extracted clusters are more sparsely connected and they start to be less well identified.

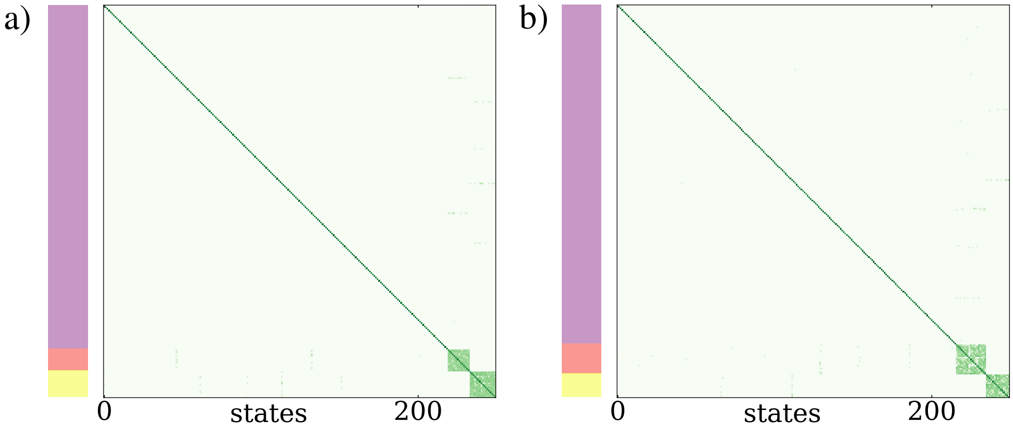

Figure S1: **Influence of the number of time steps on the simulation results**. In the results presented in the article, $3e7$ time steps were performed by the agent. **a)** Simulation with $5e6$ time steps. **b)** Simulation with $1e6$ time steps.

1.1.2 Influence of size of the proto-objects

We now investigate the performance of our algorithm when the size of the proto-objects is changed. Please recall that the spatio-temporally stable structures incorporated in the environment, referred to as *proto-objects*, consists of two overlapping squares. In the paper, these rectangles had a size of 5×5 pixels.

In Figure S2, we show the result of the Spectral clustering for proto-objects made of overlapping rectangles of size 6×6, 4×4 and 3×4, respectively. In the first two cases, the proto-objects are correctly extracted. In the last one, the size of the proto-objects is close to the size of the receptive field of the agent. They are also rather correctly extracted, although they are represented only by a very small number of centroids.

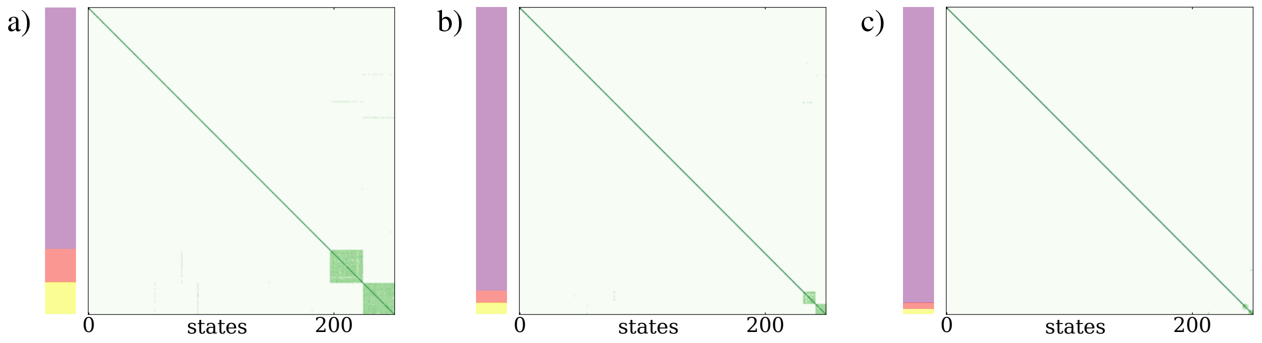

Figure S2: **Influence of the size of the proto-objects. a)** Simulation with larger objects made of two squares of size 6×6. **b)** Simulation with objects made of two squares of size 4×4. **c)** Simulation with objects made of two squares of size 3×4. In the three cases, the proto-objects are well defined, however represented by a very small number of centroids in the last one.

1.1.3 Influence of size of the environment

In Figure S3, we show the result of the simulation when the size of the environment is increased to 25×25 pixels (instead of 20×20). In this setup, two proto-objects seem to be identified, however not as precisely as in the nominal case. Indeed, a larger number of different sensory inputs are effectively received by the agent. Thus, some centroids are ambiguous in that they represent sensory states that do not come from the same structure. Increasing the number of centroids limits the part of the sensory space they represent, and thus reduces ambiguity. In that respect, it is sufficient to increase the number of $k - means$ centroids to allow for a satisfactory extraction of the proto-objects, as seen in Figure S3 c), where 400 centroids were used, but where the number of exploration was the same as in the standard case ($3e7$ steps).

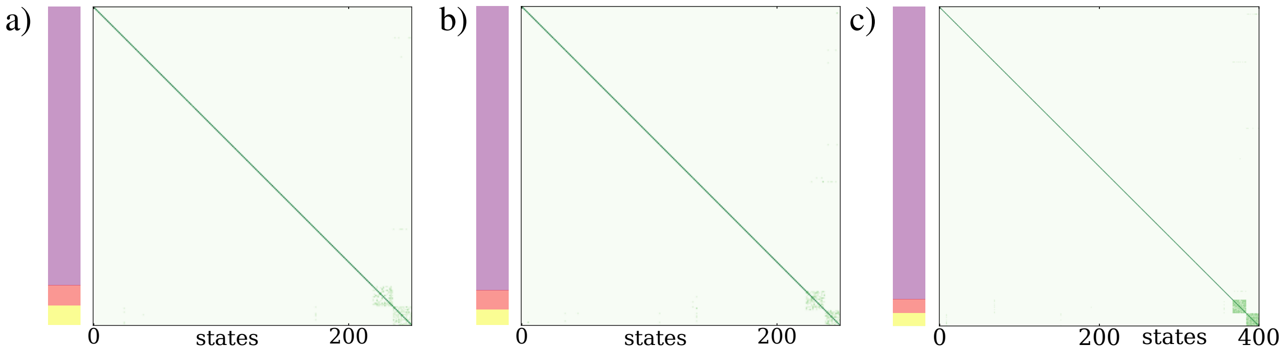

Figure S3: **Influence of the size of the environment.** In this simulation, the environment was set to a size of 25×25 pixels. **a)** Proto-objets are less well defined. **b)** Increasing the number of exploration time steps to $5e7$ does not yield a significantly better definition of the proto-objects. **c)** By raising the number of $k - means$ centroids to 400 and keeping $3e7$ exploration steps, the extraction of proto-objects is satisfactory.

1.1.4 Influence of number of-means centroids

Finally, we assess the impact of reducing the number of centroids used in the $k - means$ clustering step, and we present the results in Figure S4. Intuitively, reducing the number of $k - means$ centroids should reduce the precision of the model. While 250 centroids were used in the paper, 200 centroids are used in Figure S4 a), and 150 in Figure S4 b). In the former case, the proto-objects are correctly extracted and densely connected, but they start to be blurred in the latter, less precise setup.

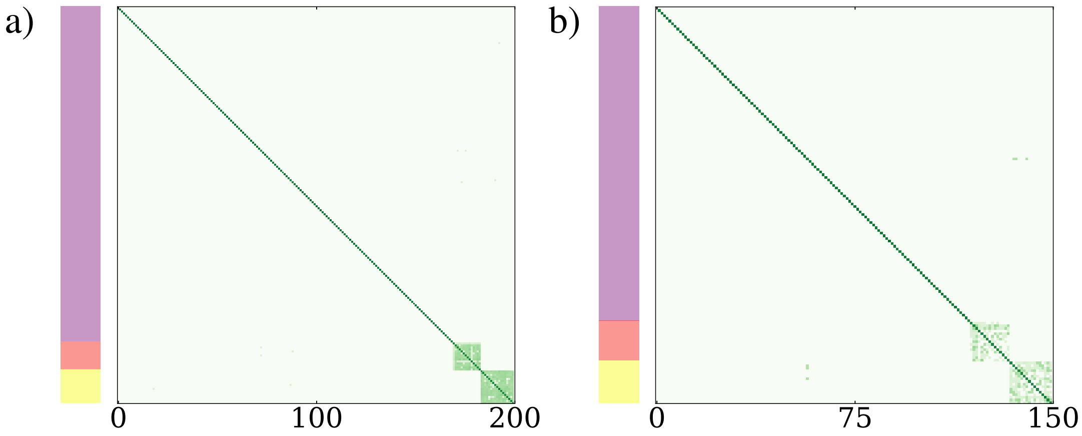

Figure S4: **Influence of the number of k-means prototypes. a)** Result of the experiment when 200 centroids are used. The extracted subgraphs are correctly extracted and densely connected. **b)** Result of the experiment using 150 centroids. The proto-objects seem to be blurred with other sensory states.

1.2 Influence of raising the complexity of the world

In the experiments proposed in the paper, the pixels from the objects and from the environment were uniformly drawn from the set $\{0, 1, 2\}$. In Figure S5, we show the result of the simulation where the pixels are uniformly drawn between 256 values. One can see that the objects are also well defined, as in the nominal case presented in the paper.

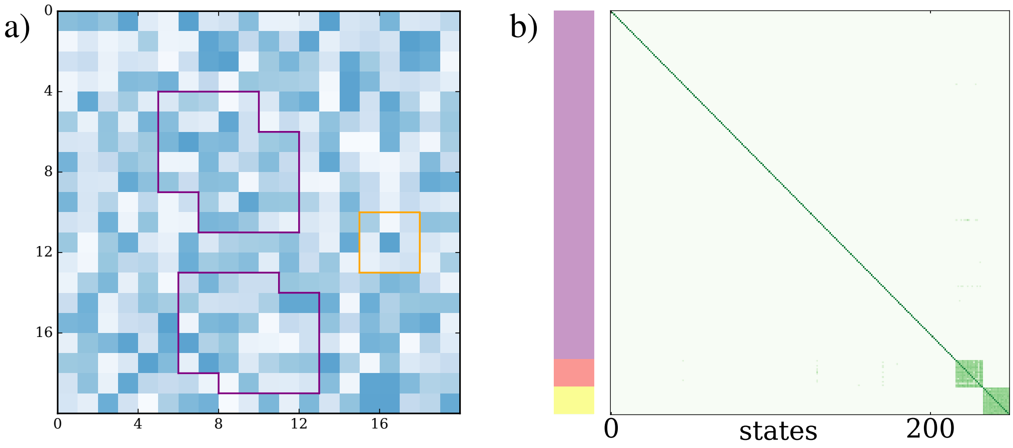

Figure S5: **Influence of the complexity of the objects and of the environment. a)** The pixels from the world and from the environment can take 256 values. **b)** Result of the Spectral Clustering in this setup.

1.3 Influence of adding randomness in the simulation

The simulation was performed in a totally deterministic environment. However, one can assess the robustness of the results to noise. In Figure S6, we show the results of the Spectral Clustering in a setup where at each time step, the sensory input is corrupted by a normal centered noise of standard deviation σ. Each input pixel is corrupted with the same amount of noise. This experiment is intended to assess the effect of a global and external parameter that influences the whole visual scene. In a real world setup, this parameter could for instance be the global lighting that randomly changes in time.

When the standard deviation is $\sigma = 0.2$ (which means 20 percents of the difference between two uncorrupted pixel values), the proto-objects are as well identified as in the paper case (Figure S6 a)). When the standard deviation is $\sigma = 0.4$, the agent is not able to extract proto-objects anymore.

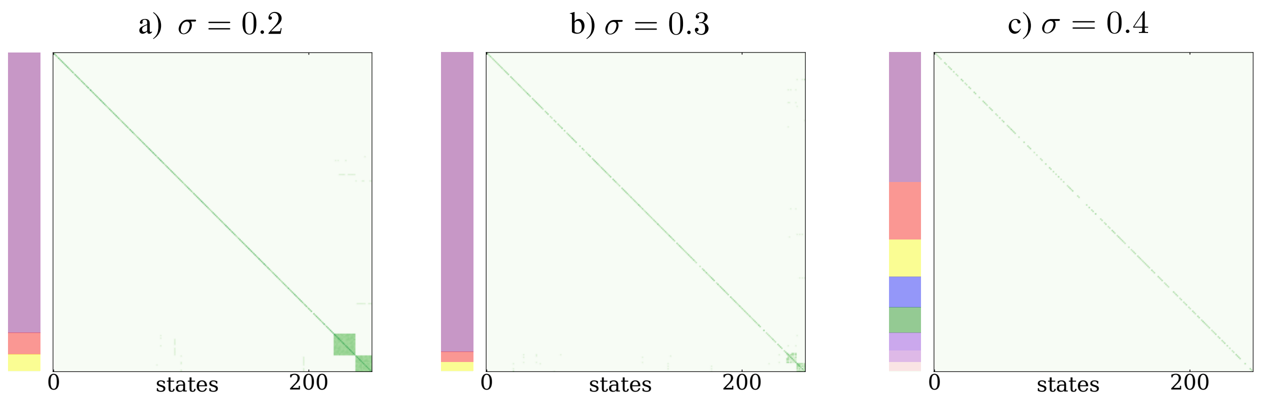

Figure S6: **Influence of adding randomness to the algorithm by corrupting the sensory inputs with a normal noise. a)** Result when introducing a normal noise of standard deviation $\sigma = 0.2$. **b)** Result when a normal noise of standard deviation $\sigma = 0.3$ is introduced. **c)** Result when a normal noise of standard deviation $\sigma = 0.4$ is introduced. The proto-objects are not correctly extracted.

1.4 Adding randomness and complexity

In this simulation, we simply investigate the impact of adding more randomness in the simulation and raising its complexity at the same time. In Figure S7, the pixels can take 256 values, and a global random noise of standard deviation σ_{global} is incorporated in the simulation, as in 1.3. Furthermore, another normal noise independently corrupts the individual pixels of the sensor of the agent, at each time step. This accounts for a measurement noise with standard deviation σ_{local}. We can see that the extraction of proto-objects is robust to perturbations with a standard deviation that is smaller than 20.

1.5 Frequency of sensory states

This simulation was motivated by a baseline experiment suggested by one of the reviewers. Another way to perform proto-object discovery would be to assess which sensory states are most frequently encountered by the agent, and manually investigate which of them are consistently linked by small movements, hopefully to extract two densely connected subgraphs.

In Figure S8, we show the distribution of the number of times each sensory state is experienced. It is interesting to divide sensory inputs in two categories: indeed, it is possible to manually attribute a $k - means$ centroid to each sensory input encountered while exploring the actual proto-objects. Each of

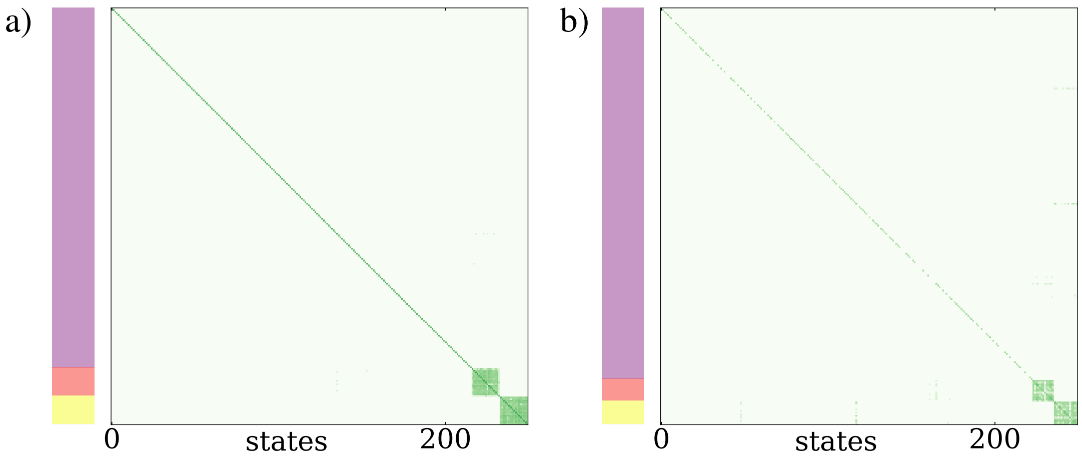

Figure S7: **Influence of combining a more complex simulation and more randomness to the setup. a)** Spectral Clustering with corruptions of standard deviation $\sigma_{global} = \sigma_{local} = 10$. **a)** Spectral Clustering with corruptions of standard deviation $\sigma_{global} = \sigma_{local} = 20$.

these centroids is colored in red, while all other centroids are colored in blue. Interestingly, we can see that some of the most frequently received sensory states are not part of a proto-object.

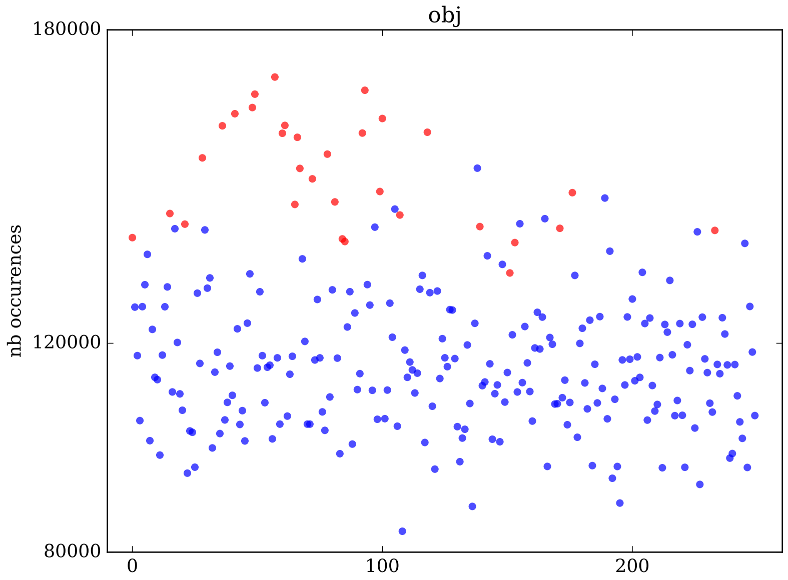

Figure S8: **Distribution of the number of occurrences of each sensory state during the simulation.** The states that correspond to an actual input coming from a proto-object are colored in red. All other states are colored in blue. Some of the most frequently encountered sensory inputs are not part of an object.

1.6 Detection of the probability threshold

In Section 2.2.2 of the paper, we describe the way the sensorimotor similarity is built by setting a threshold $p_{sim} = 0.3$ to discriminate sensorimotor transitions that come from the structure induced by the presence of proto-object from the transitions that come from randomness in the environment. This

value could seem arbitrary. However, we give a justification to the choice of this value in Figure S9. In Figure S9 a) the histogram of the conditional probabilities shows that the global distribution is divided in two main groups, with probabilities greater or smaller than a value corresponding to the minimum of the histogram, around 0.3. In Figure S9 b), we investigate how these values relate to the structure induced by the presence of proto-objects, by dividing the transitions in three categories. The transitions between two sensory inputs of a proto-objects are colored in red, while transitions between an input from a proto-object and an environment state are in orange. All other transitions are colored in blue. It appears that the group of transitions that are above 0.3 correspond to the transitions coming from the proto-objects. Thus, the value 0.3 is also grounded in the data. Based on this result, it might be possible in future work to implement an automatic criterion to choose an optimal value for this threshold p_{sim}.

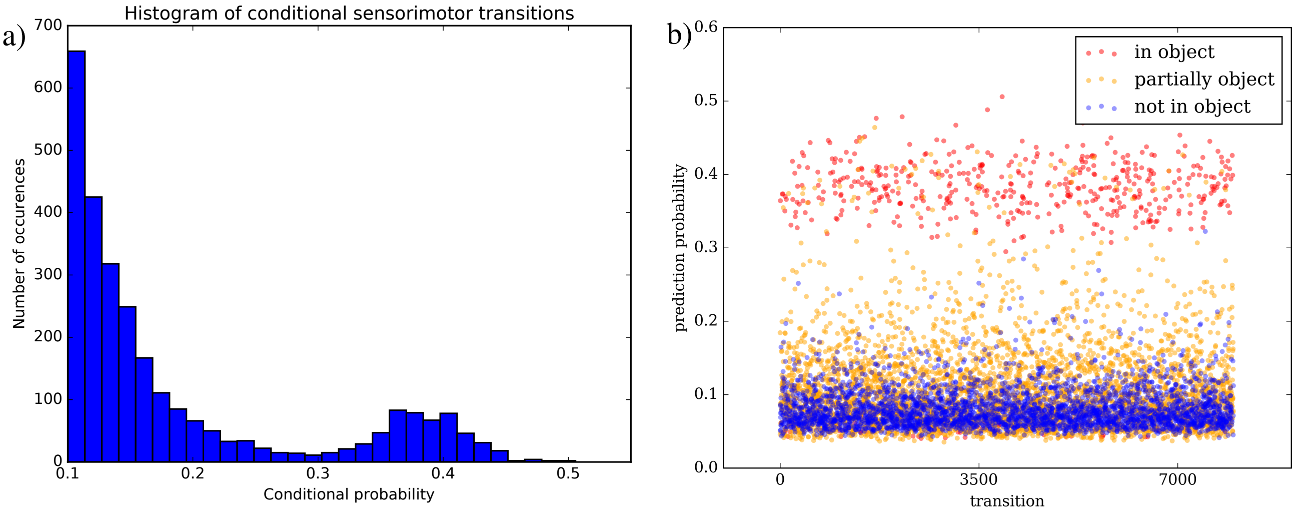

Figure S9: **Distribution of the probabilities of sensorimotor transitions. a)** Histogram of the distribution of the sensorimotor transitions. The transitions with a probability below 0.1 are filtered out in order to enhance the visualization of the minimum in the distribution. **b)** The transitions can be divided in three categories. The most probable ones, in red, come from the presence of the proto-objects.
